# Changes in dietary patterns from preconception to during pregnancy and its association with socio-demographic and lifestyle factors

**DOI:** 10.1017/S136898002100450X

**Published:** 2022-09

**Authors:** Dereje G Gete, Michael Waller, Gita D Mishra

**Affiliations:** Centre for Longitudinal and Life Course Research, School of Public Health, Faculty of Medicine, University of Queensland, 266 Herston Road, Brisbane, QLD 4006, Australia

**Keywords:** Dietary pattern changes, Preconception, Pregnancy, Factor analyses, Healthy eating index-2015 score

## Abstract

**Objective:**

To examine dietary patterns changes from preconception to during pregnancy and their associations with socio-demographic and lifestyle factors.

**Design:**

This study used data from the Australian Longitudinal Study on Women’s Health (ALSWH), a population-based prospective cohort study. Women’s dietary patterns were assessed using Healthy Eating Index-2015 (HEI-2015) score and the four patterns were obtained from the factor analysis (Western diets, vegetable and grains, traditional vegetable and fruit patterns). Multi-variable linear regression and repeated measures mixed-effect models were used.

**Setting:**

A national representative survey which covers all Australian citizens and permanent residents in Australia.

**Participants:**

621 women were included from the ALSWH.

**Results:**

Women’s scores increased on the ‘HEI-2015’, ‘traditional vegetable’ and ‘fruit’ patterns while the ‘vegetable and grains’ decreased from preconception to during pregnancy. Women with higher education were more likely to increase their HEI-2015 score and fruit consumption from preconception to during pregnancy, respectively (*β* = 2·31, (95 % CI 0·02, 4·60)) and (*β* = 23·78, (95 % CI 4·58, 42·97)), than those with lower educational status. Single women were more likely to increase the consumption of vegetables and grains compared to married women (*β* = 76·08, (95 % CI 20·83, 131·32)). Women with higher income had a greater increase in the HEI-2015 score than those with lower income (*β* = 3·02, (95 % CI 0·21, 5·83)).

**Conclusion:**

The findings indicate that there have been marked dietary changes from preconception to during pregnancy. Changes in healthy dietary patterns were influenced by education, marital status and income.

Establishing healthy dietary patterns is crucial during preconception and pregnancy periods, which have a significant role in maternal and child health^(1–4)^. The fetal development process requires the correct quality and quantity of nutrients. Altered maternal nutrition during key developmental stages might have a permanent effect on developing fetal tissues, termed as ‘programming’, and represents a pertinent risk factor for chronic and metabolic diseases in adulthood, including diabetes and cardiovascular diseases^(5,6)^. The transition in diets from preconception to during pregnancy might be affected by maternal socio-demographic and lifestyle factors^(7)^. Examining women’s dietary patterns during the transition into pregnancy is important to provide information on which type of diets change over time and why, how and when the dietary changes occur.

Recently, researchers have shown an increased interest in using the dietary patterns approach to assess women’s dietary consumption since a single nutrient or food approach has several conceptual and methodological limitations^(8,9)^. Three approaches have been widely used to explore dietary patterns: a posteriori (factor and cluster analysis), priori (dietary indices) and hybrid methods (reduced rank regression)^(10)^. A number of studies used data-driven methods, such as factor analysis to identify dietary patterns at several time points and examined the stability and reproducibility over time^(11–13)^, however, this has some limitations^(14)^. Dietary indices are constructed based on predefined dietary recommendations or previous knowledge of ‘healthy diets’, including Healthy Eating Index-2015 (HEI-2015) and Mediterranean diet score^(10)^. The HEI assesses dietary quality rather than quantity, which aligns with the Dietary Guidelines for Americans^(15)^.

The methods for characterising changes in dietary patterns over time are relatively new and are still in development. Over the last decade, two methods have been commonly used to describe changes in dietary patterns over time: applied score and natural score. Many researchers chose applied score, calculated by multiplying each individual’s consumption at a follow-up time point, which was standardised to the mean and sd of a baseline time point, by the coefficients from the principal component analysis or factor analysis at the initial phase^(11–13,16,17)^. The natural score has also been used in several prospective studies, which is calculated using coefficients obtained from the data at the follow-up phase^(18–20)^. Crozier *et al*. have compared applied and natural scores, concluding that applied scores are more appropriate to characterise changes in dietary patterns over time since the scale of measurement remains constant^(17)^. Northstone *et al*. recommended the use of a natural score in their study due to the difference in FFQ between the two-time points^(20)^.

Several longitudinal studies have examined changes in dietary patterns overtime at a population level by conducting separate factor analyses at each time point^(11,12)^. Recently, there has been a growing interest in examining the association between the changes in dietary patterns over time and disease risk^(21,22)^. However, there is little published data on changes in dietary patterns from preconception to during pregnancy. No studies to date have explored predictors of dietary pattern changes from preconception to during pregnancy. Crozier *et al.* reported that there was little change of dietary patterns from preconception to during pregnancy^(17)^. Cuco *et al*. also found no significant change in dietary patterns from preconception to during pregnancy and showed that this lack of change persisted at 6 months of postpartum^(7)^.

This study set out to examine changes in dietary patterns from before to during pregnancy and association with socio-demographic and lifestyle factors using data from a nationally representative longitudinal study of Australian women. We further examined whether the changes in dietary patterns differed between the women who were pregnant at the follow-up phase and the women who were not, by testing for an interaction.

## Methods

### Study participants and design

This study utilised data from the Australian Longitudinal Study on Women’s Health (ALSWH), an ongoing large longitudinal population-based prospective cohort study investigating factors affecting women’s health and well-being. The ALSWH started in 1996, and over 14 000 women born in 1973–1978 (aged 18–23 years) were recruited for this study. A large number of women did not respond between the baseline survey and Survey 3 (*n* 4606). Any potential biases introduced by attrition or loss of participants were shown not to influence meaningful longitudinal study^(23)^ through the attrition was high between Surveys 1 and 3 (*n* 5166). The study subjects were randomly selected from the National Universal Health Insurance database (Medicare) and the data were collected every 3 years interval from 1996 to 2015. Further details of the ALSWH have been published previously^(24)^.

**Fig. 1:**
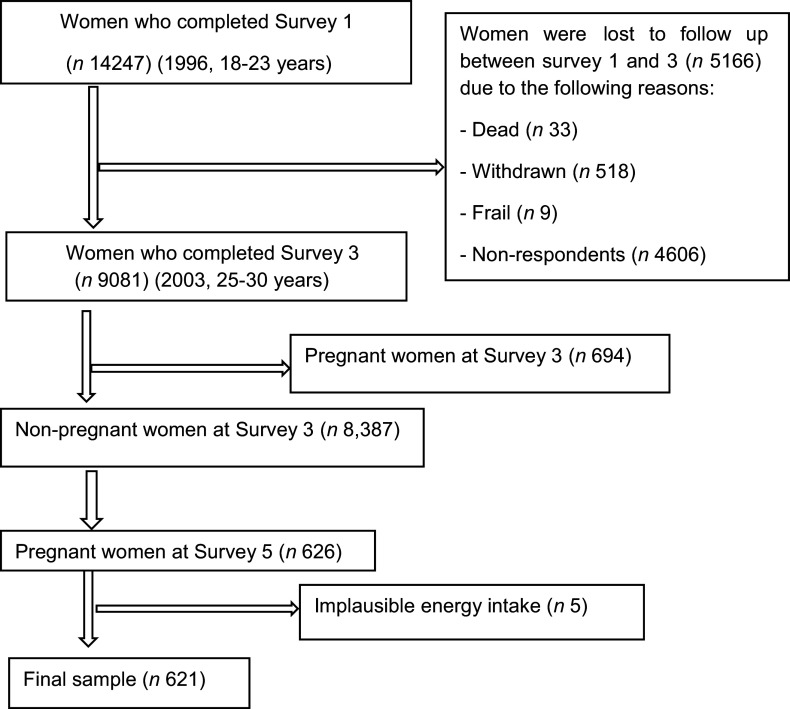
Flow diagram of the final sample for the analysis of changes in dietary patterns from preconception to during pregnancy and its association with socio-demographic and lifestyle factors between Surveys 3 and 5

The ALSWH collected women’s dietary information in 2003, Survey 3, aged 25–30 years (*n* 9081) and in 2009, Survey 5, aged 31–36 years (*n* 8199). This study included women who were non-pregnant and nulliparous at the initial phase, Survey 3 (2003) and pregnant at the follow-up phase, Survey 5 (2009), *n* 626. Women who had implausible energy intake (> 16 800 kJ/d or < 2100 kJ/d, *n* 5) were excluded^(25)^. We included a total of 621 women in the final sample for the analyses of changes in dietary patterns from preconception to during pregnancy and associated risk factors (Fig. 1).

### Dietary assessment

Women’s dietary intake was assessed using the Dietary Questionnaire Epidemiologic Study version 2. Women were asked to report their habitual dietary intake of the previous year using a validated and semi-quantitative 101-item FFQ. The dietary information was first collected at Survey 3 (2003) and the follow-up phase, Survey 5 (2009). The evaluation and development of FFQ have been described elsewhere^(26)^. The FFQ was validated for sixty-three women of child-bearing age against 7-d weighed food records who participated in an iron deficiency study^(27)^.

Women’s dietary patterns based on 101 food items (g/d) were identified using factor analysis with the use of orthogonal (varimax) rotation. The HEI-2015 score was also used to assess women’s diet quality, which is the latest diet quality index and was designed to align with the dietary guidelines for Americans. The HEI-2015 score comprises thirteen food components that sum to a total maximum score of 100 points. The food components are calculated on a density basis out of 1000 calories except for fatty acids^(15,28)^. Nine food components, including total fruits, whole fruits, total vegetables, whole grains, greens and beans, total proteins, dairy, seafood/plant proteins and fatty acids were to be consumed adequately, in which women with higher intakes receive higher scores. These food components are rich in a wide variety of nutritious foods, women with higher consumptions reduce the risk of diet-related chronic disease and promote health and wellbeing. However, four food components, such as saturated fats, added sugars, refined grains and sodium were to be consumed in moderation, in which women with lower consumptions receive higher scores. A maximum score of HEI-2015 is 100 points showing perfect adherence.

### Assessment of socio-demographic and lifestyle factors

This study assessed a number of socio-demographic and lifestyle factors and pregnancy complications, including age, marital status, education, area of residence, self-income, smoking, alcohol intake, pre-pregnancy BMI (all from Survey 3), hypertensive disorder in pregnancy and gestational diabetes mellitus (both from Survey 5). The area of residence was classified as urban and rural/remote areas^(29)^. The smoking status was categorised as never smoker, ex-smoker and current smoker^(30)^. The alcohol consumption was categorised as a non-drinker, low-risk drinker (≤14 drinks per week), risky drinker (15–28 drinks per week) and high-risk drinker (>28 drinks per week) based on the classifications of the National Health and Medical Research Council in Australia^(31)^. Only two women were high-risk drinkers (0·32 %), so this was combined with the risky drinker group. Physical activity was derived from total metabolic equivalent (MET) values based on frequency and duration of walking and moderate and vigorous intensity activity and categorised as sedentary/low (<600 MET min/week), moderate (600 to 1200 MET min/week) or high (≥1200 MET min/week)^(32)^. Women were asked whether they had been diagnosed or treated for hypertensive disorder in pregnancy and gestational diabetes mellitus at the follow-up phase (Survey 5).

### Statistical analyses

Statistical analysis was performed using SAS software version 9.4 (SAS Institute Inc.) and Stata software version 16 (StataCorp.). We used the HEI-2015 score and factor analyses with orthogonal (varimax) rotation to explore women’s dietary patterns. The number of dietary patterns was selected based on eigenvalues > 2, the identification of a breakpoint in the scree plot and factor interpretability^(33)^. We used the Kaiser–Meyer–Olkin test (0·65) to measure sampling adequacy. Food items with factor loadings > 0·30 on a factor were considered to have a strong association with that dietary pattern and be the most explanatory in describing the factors^(14)^. We ran factor analysis separately for preconception (Survey 3) and pregnancy period (Survey 5), and the factors identified were similar in relation to the number of dietary patterns and the food items that loaded highly, so the factor loadings based on the Survey 3 analysis were used. To compare each dietary pattern at the two-time points, we generated dietary patterns scores by summing up each food item (g/d) that most heavily contributed to the pattern (factor loading > 0·30). These g/d calculations were calculated at the Surveys 3 and 5 timepoints to allow comparison of daily consumption over time. This produced a more practical and interpretable measure of consumption. Our main outcome variables were changes in each dietary pattern in grams per day from preconception to during pregnancy, where the score for totals for each pattern at the initial phase (Survey 3) was subtracted from the totals at the follow-up phase (Survey 5). Spearman’s correlation coefficient was used to measure the stability of dietary pattern scores from preconception to during pregnancy. A paired *t*-test was used to assess the change in the mean dietary pattern scores at the two-time points. We checked data normality and skewness using histogram and Kolmogorov–Smirnov test. We also checked the normality of the distribution of the residuals and their variance. The distributional assumptions of the outcomes and the residuals for linear regression were met. Pearson’s correlation, *t*-test and ANOVA were used to describe the association between women’s dietary patterns score and socio-demographic and lifestyle factors. A multi-variable linear regression model was computed to examine the influence on changes in dietary pattern score of a variety of initial phase factors, including age, marital status and education, area of residence, self-income, smoking, alcohol intake, BMI, hypertensive disorder in pregnancy and gestational diabetes mellitus. We retained the socio-demographic and lifestyle factors in the adjusted model if the *P*-value was < 0·2 in the bivariate model. Analyses were also conducted to assess the stability and changes in dietary patterns from Surveys 3 to 5 in non-pregnant women (*n* 6142). A repeated measure mixed model was used to observe whether the changes in dietary patterns differed between the women who were pregnant at Survey 5, and the women who were not, by testing for an interaction. Multi-collinearity was assessed using the variance inflation factor/tolerance. We further ran a paired *t*-test and Spearman’s correlation test to observe the stability and mean changes in each food component identified by factor analyses as well as the HEI-2015 score at the two-time points. The model adequacy was checked by using the likelihood ratio test and *R* ^2^ test. The strength of association was assessed in terms of statistical significance, effect size and percentage of variance. *P*-value ≤ 0·05 was considered statistically significant.

## Results

The primary analysis was conducted using 621 women from the ALSWH as shown in Fig. 1. The mean age of women before and during pregnancy was 27 (sd 1·4) and 33 (sd 1·4) years, respectively. Of the pregnant women, 117 (18·8 %), 250 (40·3 %) and 254 (40·9 %) were reported as first, second and third trimesters, respectively. Over 85 % of women were nulliparous at the initial survey, ∼8 % were primiparous and 3 % were multiparous. ∼22 % of women had sometimes symptoms with difficulty of sleeping. The majority of women (42 %) were professional, including doctor, nurse, teacher, artist, etc.).

As can be seen from Table 1, four dietary patterns were identified from factor analyses in Surveys 3 and 5 with eigenvalues > 2 from scree plot and factor loadings. They explained 40 % of the total variation in food intake at both surveys. The first pattern was labelled ‘Western diets’ and had high positive factor loadings for beef, chicken, sausage, cakes, potato chips, pork, bacon, lamb, meat pies, salami, pizza, fried potatoes, fried fish, pasta, chocolate, hamburger, ice-cream and sweet biscuits. The second pattern, ‘vegetable and grains’ had high positive factor loadings for mushrooms, onions, other beans, garlic, zucchini, tofu, capsicum, tomatoes, rice, pasta and spinach. The third, ‘traditional vegetable’ had positive factor loadings for pumpkins, peas, cauliflower, carrots, broccoli, green beans, cabbage, spinach, potatoes and zucchini. The fourth, ‘fruit’ had high positive factor loadings for melon, peaches, apricots, pineapple, strawberries, pears and mango. The food items of each dietary pattern and the factor loading coefficients were similar at preconception and during pregnancy.

**Table 1:** Factor loadings of food items for the four dietary patterns extracted with the use of 101 food items at preconception (Survey 3) and during pregnancy (Survey 5), *n* 621*

Western diets	Factor loading S3‡, S5§	Vegetables and grains	Factor loading S3‡, S5§	Traditional vegetables	Factor loading S3‡, S5§	Fruits	Factor loading S3‡, S5§
Beef	0·60, 0·51	Mushrooms	0·62, 0·51	Pumpkins	0·55, 0·48	Melon	0·49, 0·38
Chicken	0·54, 0·45	Onions	0·59, 0·47	Peas	0·55, 0·53	Peaches	0·44, 0·56
Sausage	0·49, 0·53	Other beans†	0·53, 0·55	Cauliflower	0·54, 0·46	Apricots	0·43, 0·43
Cakes	0·49, 0·39	Garlic	0·51, 0·42	Carrots	0·51, 0·53	Pineapple	0·43, 0·38
Potato chips	0·48, 0·37	Zucchini	0·50, 0·43	Broccoli	0·49, 0·41	Strawberries	0·40, 0·40
Pork	0·43, 0·33	Tofu	0·44, 0·37	Green beans†	0·48, 0·10	Pears	0·36, 0·29
Bacon	0·42, 0·27	Capsicum	0·41, 0·39	Cabbage	0·45, 0·37	Mango	0·35, 0·41
Lamb	0·42, 0·43	Tomatoes	0·37, 0·27	Spinach	0·41, 0·28	Yoghurt	0·35, 0·25
Meat pies	0·41, 0·31	Rice	0·37, 0·31	Potatoes	0·40, 0·47		
Salami	0·41, 0·32	Pasta	0·35, 0·20	Zucchini	0·39, 0·36		
Pizza	0·38, 0·44	Spinach	0·34, 0·41				
Fried potatoes	0·37, 0·43						
Fried fish	0·35, 0·41						
Pasta	0·34, 0·48						
Chocolate	0·32, 0·40						
Hamburger	0·32, 0·30						
Ice cream	0·32, 0·34						
Sweet biscuits	0·32, 0·34						

* Values are correlation coefficients between each food item and the dietary pattern derived from factor analysis. Absolute values <0.30 and –0.30 were not listed. Western diets, total vegetables and grains, traditional vegetables and fruits and dairy.

† Other beans: chickpeas, lentils, etc.

‡ Survey 3.

§ Survey 5.

All maternal dietary patterns had a strong correlation between preconception and during pregnancy period. However, there were significant mean changes in all dietary patterns from preconception to during pregnancy except the Western pattern diets (Table 2). The mean dietary scores substantially increased by 21·8 g/d and 9·2 g/d for the fruit and traditional vegetable patterns, respectively, while they decreased by 17·4 g/d for the vegetable and grains pattern (*P* < 0·0001). There was also a slight mean increase (0·1 points) in the HEI-2015 score between preconception and during pregnancy (*P* = 0·057). The mean increment in HEI-2015 score was highest at the first trimester of pregnancy (2·1 points). However, there was a higher mean increase observed in traditional vegetable and fruits pattern at the second and third trimesters of pregnancy, respectively. Further analyses conducted in non-pregnant women (*n* 6142), the women’s dietary scores also increased by 3·8 g/d and 7·0 g/d for the traditional vegetable and fruit patterns between Surveys 3 and 5, respectively, however, the scores decreased by 11·2 g/d for the vegetables and grain pattern (*P* < 0·0001). In repeated measures mixed model, women who were pregnant had improved their intake of traditional vegetables by 9·2 g/d compared to the preconception level. This increment was higher than that observed in non-pregnant women (3·8 g/d) over the same 6 years period (*P*-value for interaction = 0·03). Women’s scores also increased on the fruit pattern from preconception to during pregnancy by 21·8 g/d. This increase was greater than that observed in non-pregnant women (7·0 g/d) over the same 6 years period (*P*-value for interaction < 0·0001). On the other hand, women decreased their consumption of vegetables and grains between preconception and during pregnancy by 17·4 g/d. This decrement was larger than that observed in non-pregnant women over the same period (-11·2 g/d), although the test for the interaction between these two groups did not achieve statistical significance (*P* = 0·06).

**Table 2:** Changes in dietary patterns from preconception (Survey 3) to during pregnancy (Survey 5) in Australian young women*

Dietary patterns	Changes in women who were pregnant at survey 5				Changes in women who were not pregnant survey 5				Pregnancy-by-time interaction
	Time points (*n* 621)‡	Mean	sd	Spearman’s correlation coefficient (*r*)	Time points (*n* 6142)§	Mean	sd	Spearman’s correlation coefficient (*r*)	
Healthy Eating Index-2015 score	Preconception	58·40	12·50	0·41	Survey 3	56·49	12·87	0·43	
	During pregnancy	59·36	10·98		Survey 5	58·19	11·05		
	First trimester	60·48	10·88						
	Second trimester	57·86	11·67						
	Third trimester	60·32	10·17						
	Mean difference	0·96	12·67		Mean difference	1·69	12·71		
	*P*-value†	0·058		< 0·0001	*P*-value†	< 0·0001		< 0·0001	0·13
Western diets	Preconception	254·20	140·14	0·50	Survey 3	269·12	1·77	0·47	
	During pregnancy	260·59	123·26		Survey 5	266·41	1·67		
	First trimester	239·89	115·17						
	Second trimester	276·39	130·77						
	Third trimester	254·57	117·65						
	Mean difference	6·39	136·67		Mean difference	−2·71	149·48		
	*P*-value†	0·24		< 0·0001	*P*-value†	0·14		< 0·0001	0·11
Vegetable and grains	Preconception	136·83	89·30	0·50	Survey 3	133·05	91·10	0·48	
	During pregnancy	119·44	64·57		Survey 5	121·87	74·96		
	First trimester	116·02	64·52						
	Second trimester	121·03	59·96						
	Third trimester	119·45	68·99						
	Mean difference	−17·38	80·59		Mean difference	−11·17	84·86		
	*P*-value†	< 0·0001		< 0·0001	*P*-value†	< 0·0001		< 0·0001	0·06
Traditional vegetable	Preconception	92·84	60·58	0·46	Survey 3	101·36	67·48	0·51	
	During pregnancy	102·04	56·58		Survey 5	105·19	62·24		
	First trimester	91·89	48·30						
	Second trimester	106·47	61·93						
	Third trimester	102·35	54·15						
	Mean difference	9·20	59·54		Mean difference	3·84	63·36		
	*P*-value†	< 0·0001		< 0·0001	*P*-value†	< 0·0001		< 0·0001	0·03
Fruit	Preconception	116·65	95·31	0·52	Survey 3	109·72	91·75	0 44	
	During pregnancy	138·42	94·91		Survey 5	116·74	86·56		
	First trimester	131·12	90·36						
	Second trimester	126·22	97·79						
	Third trimester	153·79	92·25						
	Mean difference	21·76	98·63		Mean difference	7·02	96·81		
	*P*-value†	< 0·0001		< 0·0001	*P*-value†	< 0·0001		< 0·0001	< 0·0001

* Values are mean (sd) or correlation coefficients (*r*).

† *P*-values from the paired *t*-test, Spearman’s correlation and repeated measures mixed model to observe whether the changes in dietary patterns differed between the women who were pregnant at Survey 5, and the non-pregnant women, by testing for an interaction.

‡ Women who were pregnant at Survey 5 (*n* 621).

§ Women who were non-pregnant at Survey 5 (*n* 6142).

**Fig. 2:**
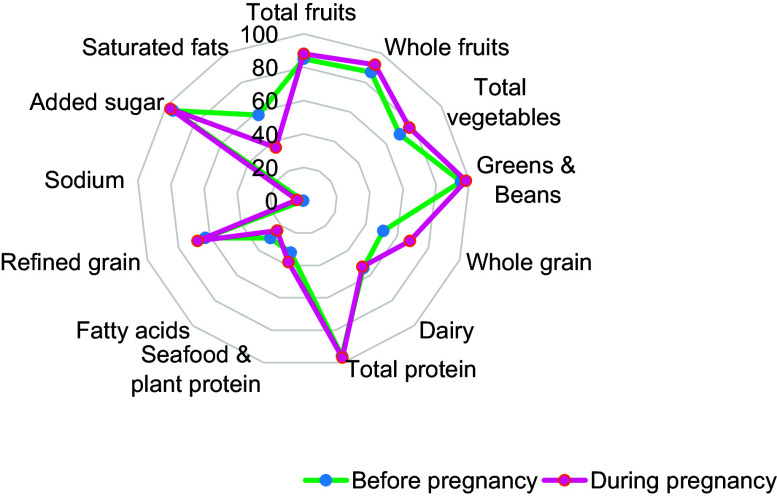
Radar plot showing the percentage of total points received for each component of the Healthy Eating Index-2015 score before and during pregnancy (*n* 621)

There was a small percentage increase in the HEI-2015 score from preconception to during pregnancy (58 % *v*. 59 %) (Fig. 2). The percentage of HEI-2015 components increased from preconception to pregnancy for the total fruits, whole fruits, total vegetables, greens and beans, whole grains, total protein, seafood and plant protein, refined grains, sodium and added sugars. However, the percentage of HEI-2015 components decreased for dairy, fatty acids and saturated fats. In both time points, women had good adherence to total fruits, whole fruits, total protein, added sugar, and greens and beans. However, they had poor adherence to sodium intake, fatty acids, saturated fats and seafood and plant protein.

Supplemental Table 1 provides mean changes in each food component from preconception to during pregnancy. Interestingly, there were significant mean differences in all HEI-2015 components except dairy and added sugar. The mean HEI-2015 components increased from preconception to during pregnancy for the total fruits, whole fruits, total vegetables, greens and beans, whole grains, total protein, seafood and plant protein, refined grains and sodium. However, the mean HEI-2015 components decreased for fatty acids and saturated fats. For meat, high fats and sugar pattern, consumption of beef, sausage, cakes, pork, bacon, lamb, pasta, chocolate, ice-cream and sweet biscuits significantly increased from preconception to during pregnancy. Within the vegetables and grain components, consumption increased slightly for only zucchini and spinach, however, the mean decreased substantially for rice, pasta, capsicum and mushrooms.

A negative correlation was found between maternal age and changes in the HEI-2015 score (the score increased from preconception to during pregnancy) (*r* = -0·08, *P* = 0·05). Younger women were more likely to improve their HEI-2015 score between preconception and pregnancy (see online Supplemental Table 2). A one-way ANOVA revealed that significant differences were observed between the category of physical activity and the consumption of HEI-2015 and fruit patterns. Women with higher physical activity (≥ 1200 MET min/week) were more likely to improve their HEI-2015 score (*P* = 0·04) and fruit consumption (*P* = 0·02) between the preconception and during pregnancy period than those with lower physical activity.

A simple linear regression model was used to assess the changes in dietary patterns from preconception to during pregnancy. This model was adjusted for the initial phase factor (dietary patterns before pregnancy) to select the candidates (*P* < 0·2) for the final model (see online Supplemental Table 3). In the multi-variable linear regression model, educational status and self-income were significantly associated with changes in the HEI-2015 score (the score increased from preconception to during pregnancy) (Table 3). Women with university/higher education had greater improvement on the HEI-2015 score than those with lower educational status (up to year 12 or equivalent) (*β* = 2·31, (95 % CI 0·02, 4·60), *P* = 0·05). Women with higher income (> 1500 $) also had a greater increase in the HEI-2015 score than those with lower income (< 999 $) (*β* = 3·02, (95 % CI 0·21, 5·83), *P* = 0·03).

**Table 3:** Socio-demographic and lifestyle factors associated with changes in dietary patterns from preconception to during pregnancy in the adjusted multi-variable linear regression model (*n* 621)

Predictors	Healthy eating index-2015 score adjusted *β*	95 % CI*	Western diets adjusted *β*	95 % CI†	Vegetables and grains adjusted *β*	95 % CI‡	Traditional vegetables adjusted *β*	95 % CI§	Fruits adjusted *β*	95 % CI‖
Women age, years	−0·58	−1·13, −0·02	5·07	−1·04, 11·18	3·10	−0·01, 6·22	–		–	
Area of residence										
Urban	0·00		0·00		0·00		0·00		–	
Rural/remote	−0·63	−2·28, 1·03	2·98	−15·01, 20·97	−8·52	−17·67, 0·63	11·45	3·14, 19·76		
Marital status										
Married	0·00		–		0·00		–		0·00	
De facto/separated/divorced	−0·25	−2·50, 1·99			−0·87	−13·15, 11·41			4·96	−14·19, 24·11
Single	−5·14	−14·82, 4·54			76·08	20·83, 131·32	–		61·94	20·28, 144·17
Educational status										
Up to year 12 or equivalent	0·00		0·00				0·00		0·00	
Trade/apprenticeship/certificate/diploma	0·69	−1·86, 3·25	−38·21	−65·89, −10·54			−7·52	−20·15, 5·11	18·13	−3·59, 39·85
University/higher degree	2·31	0·02, 4·60	−29·34	−54·67, −4·01	–		−7·30	−18·46, 3·86	23·78	4·58, 42·97
Smoking status										
Never smoked	–		0·00		0·00				0·00	
Ex-smoker			−4·89	−30·01, 20·22	−11·25	−23·83, 1·32	–		−21·43	−40·76, −2·11
Current smoker			10·52	−13·35, 34·40	−4·92	−16·84, 7·01			−6·17	−24·71, 12·36
Alcohol intake										
Non-drinker	0·00		0·00							
Rarely drinker	−3·14	−6·83, 0·54	6·75	−32·99, 46·49	–		–		–	
Low-risk drinker	−1·57	−4·95, 1·81	4·14	−32·51, 40·80						
Risky drinker	−3·60	−8·86, 1·65	26·21	−31·24, 83·66						
Physical activity										
Sedentary/low, < 600 metabolic equivalent (MET) min/week									0·00	
Moderate, 600–1200 MET min/week	–		–		–		–		−14·13	−31·63, 3·38
High, ≥ 1200 MET min/week									3·75	−11·82, 19·32
Pre-pregnancy BMI	–		1·87	0·02, 3·72	–		0·78	−0·05, 1·61	–	
Self-income (weekly)										
< 999 $	0·00		0·00		–		0·00		–	
1000 $–1499 $	2·54	0·49, 4·60	−9·59	−31·93, 12·76			−3·88	−14·09, 6·33		
>1500 $	3·02	0·21, 5·83	−17·63	−47·75, 12·50			−9·07	−22·88, 4·74		
Do not know/do not want to answer	1·79	−1·78, 5·37	−11·53	−51·29, 28·23			−21·70	−39·90, −3·50		
Gestational diabetes mellitus (GDM)										
No	–		0·00		–		0·00		–	
Yes			−42·31	−88·94, 4·32			−22·08	−42·78, −1·38		
Hypertensive disorder in pregnancy										
No	0·00		0·00		–		–		–	
Yes	−0·57	−3·79, 2·64	30·38	−4·88, 65·65						
Pre-pregnancy dietary pattern	−0·66	−0·73, −0·59	−0·60	−0·67, −0·54	−0·65	−0·70, −0·60	−0·58	−0·65, −0·52	−0·56	−0·63, −0·49

* Adjusted for maternal age, area of residence, marital status, educational status, alcohol intake, self-income, gestational hypertension and Healthy Eating Index-2015 score before pregnancy.

† Adjusted for maternal age, area of residence, educational status, smoking status, alcohol intake, pre-pregnancy BMI, self-income, GDM, hypertensive disorder in pregnancy, meats, high fats and sugar pattern before pregnancy.

‡ Adjusted for maternal age, area of residence, marital status, smoking status and vegetables and grain pattern before pregnancy.

§ Adjusted for an area of residence, educational status, self-income, pre-pregnancy BMI, GDM and traditional vegetable pattern before pregnancy.

‖ Adjusted for educational status, marital status, smoking status, physical activity and fruits and dairy pattern before pregnancy.

We adjusted different socio-demographic and lifestyle factors for each dietary pattern score. We also adjusted for the consumption of the food types at baseline. The blanks indicate unadjusted independent variables.

From dietary patterns identified by factor analyses, educational status was significantly associated with changes in Western diets (the score increased from preconception to during pregnancy) (Table 3). Women with university/higher education had a 29 g/d lower change in the Western diets than those with lower educational status (up to year 12 or equivalent) (*β* = −29·34, (95 % CI −54·67, −4·01), *P* = 0·02). In contrast, educational status was positively associated with changes in fruit pattern (the score increased from preconception to during pregnancy) (*P* = 0·01). Women with university/higher education increased their fruit consumption by 24 g/d more than those with lower educational status (up to year 12 or equivalent) (*β* = 23·78, (95 % CI 4·58, 42·97)). There was also a significant association between marital status and changes in vegetables and grains pattern (the score decreased from preconception to during pregnancy) (*P* = 0·007). Single women increased their consumption of vegetables and grains by 76 g/d more than married women (*β* = 76·08, (95 % CI 20·83, 131·32)) between preconception and pregnancy. Women living in rural/remote areas increased their consumption of vegetables by 11 g/d more than those living in urban areas (*β* = 11·45, (95 % CI 3·14, 19·76)) at *P* = 0·007.

We further examined changes of weight and energy intake from preconception to during pregnancy (see online Supplemental Table 5). Women had a significant weight gain during pregnancy by 2·7 kg (sd 7·0), *P* < 0·0001. The mean weight of women before and during pregnancy was 65·8 (sd 13·3) and 68·5 (sd 14·1) kg, respectively. There was also a significant mean difference in energy intake from preconception to during pregnancy. The women’s energy intake increased from preconception to during pregnancy by 648 kJ/d, *P* < 0·0001.

## Discussion

This study set out with the aim of examining changes in dietary patterns from preconception to during pregnancy and association with socio-demographic and lifestyle factors. In this population-based longitudinal study, we found marked mean changes in dietary patterns from preconception to during pregnancy. The women’s dietary scores increased on the HEI-2015, traditional vegetable and fruit patterns while the vegetable and grains decreased from preconception to during pregnancy.

This finding is contrary to that of Crozier *et al*. (2009) who found that a minimal decrease in the prudent diet score during early pregnancy, however, a slight mean increase in high-energy diet score in late pregnancy^(17)^. Our finding also contradicts a previous study conducted by Cuco *et al*. (2006) who reported no significant change in dietary patterns from preconception to during pregnancy, that persisted at 6 months of postpartum^(7)^. Using data from ALSWH, Hure *et al*. showed that women did not appear to improve the quality of their diets when planning to become pregnant or during pregnancy^(34)^. This inconsistency may be due to the methods to describe changes in dietary patterns score over time, for example, Crozier *et al*.^(17)^ chose applied score, basing dietary scores at a follow-up time point on patterns determined by principal component analysis at a baseline time point. Cuco *et al*.^(7)^ and Hure *et al*.^(34)^ did not use any methods to characterise changes in dietary patterns over time. The discrepancy might also be attributed to extraction methods of dietary patterns and the number of dietary patterns identified, for example, Hure *et al*.^(34)^ used the Australian Recommended Food Score to measure women’s diet quality.

We also observed similar results in non-pregnant women (*n* 6142). The non-pregnant women’s dietary scores increased from Surveys 3 to 5 for the traditional vegetable and fruit patterns, however, their scores decreased for the vegetable and grain pattern. These mean changes in each of these dietary patterns were lower than those observed in the sample who became pregnant over the same 6 years period.

In this study, women appeared to consume higher-quality diets in pregnancy than in the preconception period. This finding indicates that women gave much attention to the quality of diets during pregnancy. However, a preconception diet has a significant role in fetal and placental tissue developments, with the baby fully formed by the end of the 12th week of gestation^(35–37)^. The mean dietary scores declined from preconception to during pregnancy for the total vegetable and grains pattern. This reduction might be due to a substantial decrease in refined grain components in pregnancy, such as pasta and rice. However, there was a small mean increase in the total vegetable components during pregnancy.

Educational status was significantly associated with changes in HEI-2015 score, Western diets and fruit patterns. These findings indicate that women with higher education were more likely to increase the consumption of healthy diets during pregnancy than those with lower educational status. Education has a significant role to improve women’s nutrition knowledge, attitudes toward nutrition, diet quality, lifestyle and these might influence dietary behavioral changes^(38)^.

Another important finding was that self-income was positively associated with changes in the HEI-2015 score. Women with higher income were more likely to increase the consumption of healthy diets from preconception to during pregnancy. This may be explained by women with higher having improved access to quality foods, that is, higher consumption of a wide variety of nutritious foods, such as fruits, vegetables, whole grains and protein diets^(39,40)^.

Single women were more likely to increase consumption of vegetable and grains pattern from preconception to during pregnancy as compared to married women. This suggested that single women were more likely to shift to more healthy dietary practices compared to married women. Previous studies showed that marriage and cohabitation were associated with better adherence to healthy diets, which might be caused by more regular or formalised shopping and dietary habits when people start living together^(41,42)^. However, it is thought that women might have benefitted from this transition less than men since women are more likely to take responsibility for the diets of family members^(42,43)^.

Women living in rural/remote areas were more likely to increase in traditional vegetable patterns from preconception to during pregnancy as compared to those living in urban areas. This association might be due to the regional differences in vegetable consumption, which is higher in rural areas than in urban areas. Keeping vegetable cultivation in rural areas contributes to the high consumptions of vegetables of rural residents^(44)^.

We found wide CIs on women’s education, marital status and residence in the multi-variable analyses, these might be due to the small sample size. Further well-powered longitudinal studies are, therefore, are an essential next step to confirm the findings.

For pregnant women, the dietary guidelines advise consuming high fibre diets, such as vegetables, fruit, whole grains and legumes. These diets can reduce constipation which is a common symptom during pregnancy^(45,46)^. In this study, women improved their consumption of these high fibre diets from preconceptions to during pregnancy. However, they had poor adherence to wholegrains at both time points. The guidelines also recommend avoiding consuming foods associated with increased risk of *Listeria* bacteria, including cold seafood, soft cheeses, bean sprouts and packaged salad^(45)^. These diets were not highly loaded in the factor analyses. However, the mean of HEI-2015 components increased for the seafood and plant proteins, while decreased for the dairy from preconception to during pregnancy in our study.

The main strengths of this study are the population-based prospective cohort study and comprehensive information on women’s socio-demographic and lifestyle factors. Another advantage is using both posteriori (factor analyses) and priori (HEI-2015 score) approaches to identify women’s dietary patterns. The factor analysis is the most popular approach in the data reduction method. The HEI-2015 score is a contemporary diet quality index where each food component is scored on a density basis out of 1000 cal. We also used a validated FFQ to assess women’s dietary intake, specifically designed for use in the Australian population. We conducted a repeated measure mixed model to examine whether the changes in dietary patterns differed between the women who were pregnant at Survey 5 and the women who were not, by testing for an interaction term. However, this study was limited by the use of self-report data on women’s diets and their characteristics, which might have information bias. The FFQ was constructed based on women’s reports of dietary consumption over the past 12 months, which could have introduced recall bias. We might also not have adequately assessed women’s dietary intake during pregnancy since the date of Survey 5 returned between March 2009 and October 2010. Although we know when the pregnancy period would have occurred in this interval, because consumption was based on the prior 12 months, some responses may have included some patterns of consumption before pregnancy. Dietary patterns might have changed to some extent in the general population since the surveys took place due to temporal trends in dieting behaviors, for example, low carb/Atkins diet. The Atkins diet, is a low-carb diet developed by Robert Atkins in the 1970s, gained widespread popularity in 2003 and 2004, for weight loss^(47)^. To compare each dietary pattern at the two-time points, we generated dietary patterns score by summing up each food item (g/d) which was loaded highly in each factor (factor loading > 0·30). Though this approach produced a more practical and interpretable measure of consumption, it may affect the overall diet quality score and alter the results as a few food items were unable to be loaded in the factors, such as rye or wholemeal bread. The findings, therefore, should be interpreted with caution.

We found a significant mean difference in dietary patterns from preconception to during pregnancy. The women’s dietary scores increased from preconception to during pregnancy for the HEI-2015, traditional vegetable and fruit patterns, while they decreased for the vegetable and grain pattern. Overall, women appeared to improve the consumption of healthy diets during the pregnancy period. Women’s education, marital status, self-income and area of residence were significantly associated with changes in healthy dietary patterns from preconception to during pregnancy. Early shaping of adequate dietary behaviors before pregnancy is very important for the mothers and their children’s health since pre-conception diets have a critical role in placental and fetal tissue developments. Further, a well-powered longitudinal study could usefully explore the stability and changes in dietary patterns at the two-time points. Strengthening nutritional advice in the healthcare setting, especially antenatal/postnatal care could be important to improve women’s awareness and attitudes towards the role of healthy diets before and during pregnancy on maternal and child health.

## Supplementary material

For supplementary material accompanying this paper visit https://doi.org/10.1017/S136898002100450X. click here to view supplementary material
