# Supplementary material for: Changes in dietary patterns from preconception to during pregnancy and its association with socio-demographic and lifestyle factors
Source: Public Health Nutr. 2021 Nov 2;25(9):2530–40. doi: 10.1017/S136898002100450X (PMC9991683; doi:10.1017/S136898002100450X)
Supplement: Supplementary file 1 [file S136898002100450Xsup001.docx]

**Changes in dietary patterns from preconception to during pregnancy and its association with socio-demographic and lifestyle factors**

Dereje Gedle Gete

Online supplementary materials

**Supplementary Table 1.** Changes of each food components from preconception to during pregnancy (n= 621) ^a^

| Dietary patterns | Food components | Before pregnancy | During pregnancy | Mean difference | *p-value ^b^* | Spearman’s correlation coefficient | *p-value ^b^* |
| --- | --- | --- | --- | --- | --- | --- | --- |
| HEI-2015 score ^c^ | Total Fruits  Whole Fruits  Total Vegetables  Greens and Beans  Whole Grains  Dairy  Total Protein  Seafood & Plant Pro.  Fatty Acids  Refined Grains  Sodium  Added Sugars  Saturated Fats | 4.26 (1.18)  4.36 (1.08)  3.51 (1.27)  4.75 (0.75)  5.07 (4.28)  5.42 (3.40)  4.79 (0.67)  1.59 (1.05)  3.04 (3.16)  6.29 (3.53)  0.03 (0.53)  9.52 (1.72)  5.76 (3.80) | 4.38 (1.08)  4.61 (0.88)  3.85 (1.14)  4.89 (0.50)  6.77 (3.80)  5.31 (3.61)  4.86 (0.50)  1.91 (1.09)  2.38 (2.96)  6.82 (3.27)  0.35 (1.72)  9.65 (1.50)  3.56 (4.17) | 0.12 (1.26)  0.26 (1.11)  0.34 (1.21)  0.14 (0.72)  1.70 (4.88)  -0.12 (3.67)  0.07 (0.65)  0.32 (1.22)  -0.65 (3.71)  0.53 (4.49)  0.32 (1.81)  0.12 (1.80)  -2.19 (5.35) | 0.02  < 0.0001  < 0.0001  < 0.0001  < 0.0001  0.42  0.008  < 0.0001  < 0.0001  0.004  < 0.0001  0.09  < 0.0001 | 0.38  0.39  0.49  0.32  0.30  0.47  0.21  0.37  0.28  0.10  0.01  0.37  0.10 | < 0.0001  < 0.0001  < 0.0001  < 0.0001  < 0.0001  < 0.0001  < 0.0001  < 0.0001  < 0.0001  0.01  0.77  < 0.0001  0.01 |
| Meat, high fats and sugar ^d^ | Beef  Chicken  Sausage  Cakes  Potato chips  Pork  Bacon  lamb  Meat pies  Salami  Pizza  Fried potatoes  Fried fish  Pasta  Chocolate  Hamburger  Ice-cream  Sweet biscuits | 35.14 (34.99)  30.73 (22.49)  6.44 (9.54)  10.89 (9.66)  3.77 (3.38)  4.56 (7.53)  2.43 (3.25)  12.04 (15.77)  12.77 (15.95)  4.11 (7.95)  19.39 (18.87)  18.85 (23.65)  5.13 (9.79)  54.12 (44.56)  10.35 (14.07)  7.88 (9.94)  9.43 (14.58)  6.14 (8.83) | 37.96 (28.92)  28.87 (21.37)  8.12 (9.60)  14.08 (15.33)  3.82 (5.74)  5.49 (7.94)  3.08 (3.24)  16.31 (16.82)  13.65 (12.77)  3.60 (6.40)  19.51 (17.50)  17.38 (18.87)  4.66 (5.99)  43.86 (31.92)  12.77 (15.02)  7.86 (9.07)  11.28 (15.07)  8.28 (9.96) | 2.82 (35.52)  -1.85(24.56)  1.68 (10.85)  3.19 (18.09)  0.05 (6.12)  0.92 (8.35)  0.65 (3.53)  4.27 (17.78)  0.88 (17.70)  -0.51 (9.28)  0.11 (20.57)  -1.47(25.11)  -0.47(10.30)  -10(43.16)  2.42 (15.35)  -0.03(10.67)  1.84 (17.49)  2.14 (10.62) | 0.05  0.06  < 0.0001  < 0.0001  0.84  0.006  < 0.0001  < 0.0001  0.21  0.17  0.89  0.14  0.25  < 0.0001  <0.0001  0.95  0.009  < 0.0001 | 0.50  0.46  0.44  0.38  0.43  0.58  0.48  0.51  0.31  0.41  0.33  0.37  0.30  0.38  0.47  0.43  0.42  0.39 | < 0.0001  < 0.0001  < 0.0001  < 0.0001  < 0.0001  < 0.0001  < 0.0001  < 0.0001  < 0.0001  < 0.0001  < 0.0001  < 0.0001  < 0.0001  < 0.0001  < 0.0001  < 0.0001  < 0.0001  < 0.0001 |
| Vegetables & grains ^d^ | Mushrooms  Onions  Other beans  Garlic  Zucchini  Tofu  Capsicum  Tomatoes  Rice  Pasta  Spinach | 5.72 (5.95)  5.92 (5.39)  2.86 (7.02)  0.63 (0.54)  7.98 (9.32)  2.24 (6.29)  3.01 (2.96)  11.55 (10.60)  38.47 (43.45)  54.12 (44.56)  4.31 (6.72) | 5.26 (5.28)  6.24 (5.27)  3.09 (5.37)  0.65 (0.49)  8.83 (8.68)  1.97 (5.58)  2.75 (2.72)  11.40 (9.93)  29.92 (29.13)  43.86 (31.92)  5.46 (7.29) | -0.46 (5.68)  0.32 (5.59)  0.24 (6.82)  0.02 (0.55)  0.84 (9.52)  -0.27 (6.71)  -0.26 (3.11)  -0.15 (11.98)  -8.56 (42.75)  -10.26(43.16)  1.15 (8.25) | 0.04  0.15  0.39  0.37  0.03  0.32  0.04  0.75  < 0.0001  < 0.0001  0.0005 | 0.59  0.46  0.59  0.48  0.52  0.56  0.49  0.37  0.36  0.38  0.46 | < 0.0001  < 0.0001  < 0.0001  < 0.0001  < 0.0001  < 0.0001  < 0.0001  < 0.0001  < 0.0001  < 0.0001  < 0.0001 |
| Traditional vegetable ^d^ | Pumpkins  Peas  Cauliflower  Carrots  Broccoli  Green beans  Cabbage  Spinach  Potatoes  Zucchini | 8.66 (10.39)  9.00 (10.94)  6.13 (7.60)  12.01 (10.34)  9.45 (8.09)  6.31 (6.88)  3.82 (7.09)  4.31 (6.72)  25.16 (26.59)  7.98 (9.32) | 10.28 (10.92)  10.23 (10.93)  6.60 (7.73)  13.74(9.74)  11.57 (8.52)  7.26 (6.67)  3.39 (5.30)  5.46 (7.29)  24.69 (24.54)  8.83 (8.68) | 1.61 (11.85)  1.23 (10.99)  0.45 (8.16)  1.73 (10.97)  2.12 (9.31)  0.95 (7.75)  -0.42 (7.09)  1.15 (8.25)  -0.48 (28.46)  0.84 (9.52) | 0.0007  0.005  0.16  0.0001  < 0.0001  0.002  0.13  0.0005  0.67  0.04 | 0.46  0.52  0.45  0.40  0.38  0.34  0.46  0.46  0.37  0.52 | < 0.0001  < 0.0001  < 0.0001  < 0.0001  < 0.0001  < 0.0001  < 0.0001  < 0.0001  < 0.0001  < 0.0001 |
| Fruits ^d^ | Melon  Peaches  Apricots  Pineapple  Strawberries  Pears  Mango  Yoghurt | 16.16 (20.86)  14.69 (24.74)  6.82 (14.15)  7.32 (11.12)  8.44 (11.12)  13.97 (21.70)  7.15 (13.62)  42.09 (51.81) | 18.51 (22.22)  14.38 (23.75)  6.76 (12.08)  7.46 (11.87)  9.81 (12.24)  22.52 (26.93)  6.51 (11.47)  52.44 (52.05) | 2.35 (24.66)  -0.31 (29.07)  -0.06 (16.41)  0.14 (14.88)  1.38 (13.28)  8.55 (28.04)  -0.6 (14.61)  10.36 (57.20) | 0.02  0.79  0.92  0.81  0.01  < 0.0001  0.27  < 0.0001 | 0.41  0.43  0.40  0.36  0.38  0.44  0.51  0.47 | < 0.0001  < 0.0001  < 0.0001  < 0.0001  < 0.0001  < 0.0001  < 0.0001  < 0.0001 |

^a^ Values are mean (SD) or correlation coefficients (r), ^b^ *p-values* from paired t-test or Spearman’s correlation. ^c^ The first 13 food components were obtained from HEI-2015 score. ^d^ The four dietary patterns obtained from factor analysis (meats, high-fats and sugar; vegetable and grains; traditional vegetable; and fruits patterns).

**Supplementary Table 2.** Women characteristics before pregnancy according to changes in dietary patterns (n= 621) ^a^

| **Characteristics** | **HEI-2015 score** | **Western diets** | **Vegetables &**  **grains** | **Traditional**  **Vegetables** | **Fruits** |
| --- | --- | --- | --- | --- | --- |
| women age, years  p-values ^b^ | -0.08  0.06 | 0.05  0.18 | 0.04  0.33 | 0.05  0.18 | 0.03  0.51 |
| Area of residence ^c^  Urban  Rural/remote  p-values ^b^ | 1.25 (12.66)  0.51 (12.75)  0.48 | 1.93 (128.66)  13.23 (148.79)  0.32 | -18.09(83.09)  -16.51(76.89)  0.81 | 8.10 (53.43)  10.57 (68.18)  0.61 | 19.18(99.52)  25.88(97.66)  0.41 |
| Marital status ^c^  Married  De facto/separated/divorced  Single  p-values ^b^ | 0.79 (12.54)  2.10 (13.05)  -4.58 (21.09)  0.43 | 9.72 (133.41)  -11.76(152.93)  24.17 (124.59)  0.35 | -16.54(78.43)  -24.28(87.49)  44.42(159.29)  0.21 | 9.45(56.54)  6.34(71.13)  46.90(119.91)  0.40 | 18.58(97.51)  35.12(100.27)  103.40(163.68)  0.32 |
| Educational status ^c^  Up to year 12 or equivalent  Trade/apprenticeship/certificate/diploma  University/higher degree  p-values ^b^ | 2.01 (14.87)  0.46 (11.52)  0.97 (12.22)  0.63 | 18.50 (146.06)  -11.12(153.45)  7.79 (124.95)  0.21 | -6.12(71.19)  -11.87(75.43)  -22.42(84.70)  0.13 | 12.15(67.64)  1.44(64.08)  10.52(55.31)  0.26 | 6.67(98.21)  30.25(91.26)  23.38(100.66)  0.17 |
| Smoking status ^c^  Never smoked  Ex-smoker  Current smoker  p-values ^b^ | 0.39 (12.07)  1.31 (12.79)  2.85 (14.86)  0.18 | 5.85 (133.14)  2.96 (126.98)  12.98 (157.04)  0.85 | -13.30 (74.18)  -28.28(79.36)  -23.68(100.12)  0.18 | 10.22(55.50)  5.92(64.14)  7.35(69.69)  0.77 | 24.25(97.52)  3.49(99.95)  26.68(102.03)  0.15 |
| Alcohol intake ^c^  Non-drinker  Rarely drinker  Low-risk drinker  Risky drinker  p-values ^b^ | 4.20 (15.10)  1.10 (13.22)  0.69 (11.92)  0.11 (17.80)  0.43 | 14.48 (148.31)  7.96 (162.81)  4.24 (123.76)  16.92 (181.92)  0.94 | -17.63(70.86)  -8.70(74.90)  -18.34(77.06)  -54.13(148.37)  0.08 | 10.32(52.86)  2.62(68.31)  11.34(57.03)  4.90(59.80)  0.51 | 30.28(92.82)  11.57(82.07)  22.50(105.07)  51.55(74.52)  0.26 |
| Physical activity ^c^  Sedentary/low, < 600 MET min/week  Moderate, 600 to 1200 MET min/week  High, ≥ 1200 MET min/week  p-values ^b^ | 2.54 (12.46)  -0.41 (13.17)  0.21 (12.45)  0.04 | -4.01 (135.69)  10.47 (136.70)  14.20 (132.85)  0.32 | -18.41(72.83)  -20.78(92.79)  -13.77(77.32)  0.68 | 6.94(62.95)  11.38(53.73)  9.28(59.18)  0.77 | 34.67(89.48)  5.55(86.37)  18.73(113.26)  0.02 |
| Pre-pregnancy BMI ^c^  p-values ^b^ | 0.02  0.59 | 0.04  0.31 | 0.03  0.42 | 0.06  0.16 | -0.04  0.30 |
| Self-income (weekly) ^c^  < 999 $  1000 $ – 1499 $  >1500 $  Don’t know/ don’t want to answer  p-values ^b^ | 0.22 (12.53)  2.66 (13.58)  0.62 (12.09)  2.94 (10.65)  0.21 | 7.28 (139.01)  1.72 (124.04)  4.55 (141.55)  9.68 (108.72)  0.98 | -16.51(80.53)  -13.33(75.60)  -34.95(91.17)  -17.07(78.27)  0.39 | 10.14(62.02)  11.20(54.32)  7.01(55.10)  -7.28(48.02)  0.42 | 22.89(98.55)  13.59(95.80)  25.14(103.74)  35.91(106.04)  0.65 |
| Gestational diabetes mellitus ^c^  No  Yes  p-values ^b^ | 0.86 (12.64)  5.34 (11.51)  0.09 | 7.13 (137.48)  -34.84(122.69)  0.14 | -16.49(79.74)  -32.90(96.95)  0.33 | 9.43(59.30)  -8.11(67.96)  0.16 | 22.00(98.83)  21.66(97.46)  0.99 |
| Hypertensive disorder in pregnancy ^c^  No  Yes  p-values ^b^ | 1.07 (12.42)  0.36 (15.29)  0.72 | 2.64 (134.29)  49.05 (161.54)  0.03 | -18.05(80.86)  1.27(60.17)  0.12 | 7.99(60.36)  20.57(48.10)  0.18 | 21.99(99.30)  22.79(93.66)  0.96 |

^a^ Values are mean (SD) or correlation coefficients (r), ^b^ *p-values* from ANOVA or t-test or Pearson’s correlation test. ^c^ missing values (area of residence: n= 2, marital status: n=4, educational status: n=19, smoking status: n=4, alcohol intake: n=3, physical activity: n=6, pre-pregnancy BMI: n= 11, self-income: n= 2, gestational diabetes: n= 8, hypertensive disorder in pregnancy: n= 10.

**Supplementary Table 3.** Socio-demographic and lifestyle factors associated with changes in dietary patterns in the crude linear regression model (n= 621)

| **Predictors** | **Healthy Eating Index-2015 score**  ^a^ Crude β (95% CI) | **Western diets**  ^a^ Crude β (95% CI) | **Vegetables and**  **grains**  ^a^ Crude β (95% CI) | **Traditional**  **Vegetables**  ^a^ Crude β (95% CI) | **Fruits**  ^a^ Crude β (95% CI) |
| --- | --- | --- | --- | --- | --- |
| women age, years | -0.44 (-0.99, 0.11) | 4.06 (-1.95, 10.07) | 2.73 (-0.37, 5.83) | 1.59 (-1.14, 4.32) | 1.08 (-3.57, 5.73) |
| Area of residence  Urban  Rural/remote | 0.00  -1.64 (-3.26, -0.03) | 0.00  13.77 (-3.87, 31.42) | 0.00  -8.89 (-18.02, 0.24) | 0.00  14.87 (6.81, 22.94) | 0.00  0.44 (-13.23, 14.11) |
| Marital status  Married  De facto/separated/divorced  Single | 0.00  -1.18 (-3.34, 0.98)  -6.46 (-16.28, 3.35) | 0.00  4.61 (-19.07, 28.29)  -9.01 (-116.69, 98.61) | 0.00  -2.06 (-14.15, 10.04)  72.68 (17.36,128.00) | 0.00  8.62 (-2.12, 19.36)  25.50 (-23.27, 74.27) | 0.00  2.61 (-15.61, 20.83)  69.87(-13.13,152.86) |
| Educational status  Up to year 12 or equivalent  Trade/apprenticeship/certificate/diploma  University/higher degree | 0.00  0.56 (-1.96, 3.08)  3.27 (1.09, 5.44) | 0.00  -43.89 (-71.04,-16.73)  -44.49 (-67.74,-21.23) | 0.00  -1.43 (-15.86, 12.99)  3.28 (-9.05, 15.61) | 0.00  -7.06 (-19.53, 5.40)  -11.80 (-22.43,-1.17) | 0.00  20.87 (-0.35, 42.09)  27.45 (9.40, 45.51) |
| Smoking status  Never smoked  Ex-smoker  Current smoker | 0.00  0.47 (-1.76, 2.72)  0.47 (-1.62, 2.57) | 0.00  -3.88 (-28.38, 20.62)  19.57 (-3.31, 42.44) | 0.00  -11.73 (-24.36, 0.89)  -5.68 (-17.45, 6.10) | 0.00  0.33 (-10.82, 11.49)  2.85 (-7.56, 13.27) | 0.00  -26.35 (-45.23,-7.47)  -11.82 (-29.51, 5.86) |
| Alcohol intake  Non-drinker  Rarely drinker  Low risk drinker  Risky drinker | 0.00  -3.30 (-6.92, 0.33)  -1.37 (-4.73, 1.98)  -4.42 (-9.47, 0.64) | 0.00  5.33 (-34.47, 45.13)  -1.94 (-38.68, 34.79)  44.77 (-10.86, 100.40) | 0.00  6.27 (-14.27, 26.81)  14.24 (-4.76, 33.23)  12.96 (-15.93, 41.85) | 0.00  -4.87 (-22.97, 13.23)  0.28 (-16.42, 16.99)  5.90 (-19.36, 31.16) | 0.00  -13.94(-44.69,16.80)  2.01 (-26.40, 30.42)  16.00 (-26.85, 58.86) |
| Physical activity  Sedentary/low, < 600 MET min/week  Moderate, 600 to 1200 MET min/week  High, ≥ 1200 MET min/week | 0.00  -0.33 (-2.40, 1.74)  0.49 (-1.34, 2.33) | 0.00  4.29 (-17.96, 26.53)  -2.41 (-22.16, 17.33) | 0.00  0.20 (-11.41, 11.81)  5.39 (-4.86, 15.64) | 0.00  3.39 (-6.83, 13.62)  1.67 (-7.36, 10.69) | 0.00  -16.76 (-34.21, 0.68)  2.57 (-12.96, 18.11) |
| Pre-pregnancy BMI | -0.05 (-0.21, 0.12) | 2.60 (0.82, 4.38) | -0.13 (-1.07, 0.80) | 1.11 (0.30, 1.93) | -0.90 (-2.29, 0.50) |
| Self-income (weekly)  < 999 $  1000 $ – 1499 $  >1500 $  Don’t know/ don’t want to answer | 0.00  3.29 (1.33, 5.25)  4.26 (1.56, 6.96)  2.80 (-0.75, 6.35) | 0.00  -20.17 (-41.57, 1.23)  -25.97 (-55.25, 3.30)  -17.46 (-56.23, 21.31) | 0.00  7.68 (-3.54, 18.91)  -5.13 (-20.50, 10.24)  7.60 (-12.75, 27.96) | 0.00  -9.14 (-18.96, 0.67)  -13.48 (-26.86,-0.10)  -26.70 (-44.41,-8.99) | 0.00  -2.74 (-19.53, 14.05)  17.39 (-5.61, 40.39)  15.79 (-14.61, 46.20) |
| Gestational diabetes mellitus  No  Yes | 0.00  2.32 (-1.74, 6.38) | 0.00  -31.38 (-75.92, 13.15) | 0.00  -8.81 (-31.92, 14.29) | 0.00  -18.38 (-38.66, 1.90) | 0.00  -1.17 (-35.72, 33.38) |
| Hypertensive disorder in pregnancy  No  Yes | 0.00  -2.37 (-5.42, 0.68) | 0.00  41.83 (8.43, 75.23) | 0.00  -1.19 (-18.64, 16.25) | 0.00  8.51 (-6.75, 23.77) | 0.00  3.62 (-22.37, 29.61) |

^a^ Crude model was adjusted for dietary patterns before pregnancy.

**Supplementary Table 4.** Factor loadings of food items for the 4 dietary patterns extracted with the use of 101 food items at preconception (Survey 3) and during pregnancy (Survey 5), n= 621 ^a^

| **Food items** | **Factor 1**  **Western diets** | | **Factor 2**  **Vegetables and grains** | | **Factor 3**  **Traditional vegetables** | | **Factor 4**  **Fruits** | |
| --- | --- | --- | --- | --- | --- | --- | --- | --- |
|  | Survey 3 | Survey 5 | Survey 3 | Survey 5 | Survey 3 | Survey 5 | Survey 3 | Survey 5 |
| **All bran** | 0.00 | -0.01 | 0.09 | 0.09 | -0.05 | 0.01 | 0.07 | -0.01 |
| **Apples** | -0.11 | 0.05 | 0.19 | 0.30 | 0.08 | 0.01 | 0.27 | 0.20 |
| **Apricots** | 0.08 | 0.07 | -0.04 | 0.10 | 0.07 | 0.01 | **0.43** | **0.43** |
| **Avocado** | 0.05 | 0.00 | 0.29 | 0.44 | 0.05 | 0.02 | 0.14 | 0.31 |
| **Bacon** | **0.42** | **0.27** | -0.09 | -0.19 | -0.12 | -0.02 | 0.09 | 0.07 |
| **Baked beans** | 0.05 | 0.00 | 0.03 | 0.05 | 0.02 | 0.09 | 0.00 | -0.01 |
| **Bananas** | -0.15 | -0.05 | 0.17 | 0.23 | -0.03 | -0.05 | 0.22 | 0.14 |
| **Bean sprouts** | -0.07 | -0.09 | 0.24 | 0.13 | 0.17 | 0.12 | 0.26 | 0.13 |
| **Beef** | **0.60** | **0.51** | -0.09 | -0.20 | 0.15 | 0.21 | 0.06 | 0.11 |
| **Beetroot** | 0.03 | 0.06 | 0.15 | 0.13 | 0.26 | 0.24 | 0.26 | 0.31 |
| **Bran flakes** | 0.08 | 0.12 | 0.11 | 0.02 | -0.08 | -0.02 | 0.02 | 0.07 |
| **Broccoli** | -0.11 | -0.13 | 0.10 | 0.03 | **0.49** | **0.41** | 0.02 | 0.01 |
| **Butter** | 0.12 | 0.07 | 0.01 | 0.15 | 0.13 | 0.12 | -0.01 | 0.03 |
| **Butter margarine blends** | 0.16 | 0.03 | 0.05 | 0.01 | -0.03 | -0.02 | -0.05 | -0.10 |
| **Cabbage** | 0.14 | 0.22 | 0.16 | 0.10 | **0.45** | **0.37** | 0.20 | 0.15 |
| **Cakes** | **0.48** | **0.39** | 0.04 | 0.10 | 0.00 | 0.06 | 0.10 | -0.01 |
| **Capsicum** | -0.13 | -0.09 | **0.41** | **0.39** | 0.00 | -0.05 | 0.06 | 0.15 |
| **Carrots** | 0.12 | 0.16 | 0.22 | 0.27 | **0.51** | **0.53** | 0.04 | 0.11 |
| **Cauliflower** | 0.03 | -0.04 | -0.14 | -0.16 | **0.54** | **0.46** | 0.06 | -0.02 |
| **Celery** | -0.08 | -0.10 | 0.16 | 0.17 | 0.06 | 0.17 | 0.29 | 0.32 |
| **Chicken** | **0.54** | **0.45** | -0.01 | -0.16 | 0.08 | 0.04 | 0.00 | 0.08 |
| **Chips** | **0.38** | **0.43** | -0.04 | -0.07 | 0.15 | 0.21 | -0.29 | -0.11 |
| **Chocolate** | **0.32** | **0.40** | 0.06 | 0.19 | -0.06 | -0.06 | -0.09 | 0.00 |
| **Cornflakes** | 0.11 | 0.17 | -0.02 | -0.07 | -0.01 | 0.04 | -0.01 | 0.01 |
| **Crackers** | 0.29 | 0.22 | 0.27 | 0.22 | -0.12 | -0.04 | 0.07 | 0.03 |
| **Cream cheese** | 0.04 | 0.16 | 0.11 | 0.21 | 0.05 | 0.07 | 0.02 | 0.01 |
| **Crisps** | **0.48** | **0.37** | 0.06 | 0.01 | 0.02 | 0.00 | -0.11 | 0.02 |
| **Cucumber** | -0.15 | -0.17 | 0.23 | 0.26 | 0.02 | -0.02 | 0.26 | 0.32 |
| **Eggs** | 0.16 | 0.11 | 0.12 | 0.22 | 0.10 | 0.03 | 0.13 | -0.01 |
| **Firm cheese** | 0.27 | 0.16 | 0.02 | -0.03 | -0.03 | 0.11 | -0.06 | 0.03 |
| **Fish** | 0.18 | 0.28 | 0.26 | 0.08 | 0.23 | 0.09 | 0.26 | 0.25 |
| **Flavoured milk drink** | 0.20 | 0.12 | -0.06 | -0.07 | -0.03 | 0.09 | -0.08 | -0.10 |
| **Fortified wines** | 0.03 | 0.09 | 0.14 | 0.09 | -0.07 | -0.10 | 0.07 | 0.00 |
| **Fried fish** | **0.35** | **0.41** | 0.12 | -0.05 | 0.10 | 0.10 | 0.02 | -0.02 |
| **Fruit juice** | 0.17 | 0.28 | 0.16 | 0.08 | 0.03 | 0.09 | -0.01 | -0.06 |
| **Full cream milk** | 0.19 | 0.20 | -0.14 | -0.16 | 0.20 | 0.31 | -0.19 | -0.11 |
| **Garlic** | -0.06 | -0.04 | **0.51** | **0.42** | 0.07 | 0.00 | -0.06 | 0.04 |
| **Green beans** | 0.00 | 0.06 | -0.11 | -0.02 | **0.48** | **0.39** | -0.01 | 0.10 |
| **Ham** | 0.28 | 0.21 | -0.07 | -0.10 | -0.05 | -0.16 | 0.05 | 0.05 |
| **Hamburger** | **0.33** | **0.30** | -0.08 | -0.21 | -0.07 | -0.03 | -0.15 | -0.04 |
| **Hard cheese** | 0.04 | 0.04 | 0.27 | 0.20 | -0.07 | -0.15 | 0.01 | 0.03 |
| **High fibre white bread** | 0.17 | 0.15 | -0.05 | -0.19 | 0.04 | 0.05 | -0.02 | 0.07 |
| **Ice cream** | **0.32** | **0.34** | 0.07 | 0.02 | 0.14 | 0.07 | 0.04 | 0.02 |
| **Jam** | 0.19 | 0.23 | 0.21 | 0.16 | 0.00 | 0.02 | 0.07 | 0.04 |
| **Lamb** | **0.42** | **0.43** | -0.13 | -0.20 | 0.08 | 0.14 | 0.04 | 0.31 |
| **Lettuce** | -0.17 | -0.19 | 0.29 | 0.30 | 0.06 | -0.08 | 0.17 | 0.23 |
| **Low fat cheese** | -0.06 | -0.03 | 0.01 | 0.06 | -0.10 | -0.09 | 0.17 | 0.09 |
| **Mango** | -0.01 | 0.05 | 0.04 | 0.02 | 0.02 | -0.06 | **0.35** | **0.41** |
| **Margarine** | 0.11 | 0.05 | -0.17 | -0.24 | -0.01 | 0.00 | -0.07 | 0.06 |
| **Meat pies** | **0.41** | **0.31** | 0.02 | -0.10 | -0.05 | -0.01 | -0.02 | -0.04 |
| **Multi grain bread** | -0.07 | 0.04 | 0.19 | 0.18 | -0.10 | -0.10 | 0.06 | 0.10 |
| **Melon** | -0.03 | 0.03 | -0.12 | -0.07 | -0.01 | -0.03 | **0.49** | **0.38** |
| **Monounsaturated margarine** | -0.03 | 0.11 | -0.07 | 0.03 | -0.01 | -0.03 | 0.00 | -0.04 |
| **Muesli** | -0.14 | -0.07 | 0.23 | 0.30 | 0.02 | -0.11 | 0.07 | 0.08 |
| **Mushrooms** | 0.04 | 0.16 | **0.62** | **0.51** | 0.19 | 0.18 | 0.02 | 0.04 |
| **Nuts** | 0.01 | 0.18 | 0.28 | 0.40 | 0.05 | -0.07 | 0.17 | 0.21 |
| **Onion** | 0.07 | 0.25 | **0.59** | **0.47** | 0.26 | 0.18 | -0.13 | 0.02 |
| **Oranges** | -0.05 | 0.00 | -0.01 | 0.10 | 0.01 | -0.09 | 0.15 | 0.22 |
| **Other beans ^b^** | -0.17 | -0.04 | **0.53** | **0.56** | 0.20 | 0.04 | 0.08 | 0.14 |
| **Pasta** | **0.34** | **0.48** | **0.35** | **0.21** | -0.03 | 0.00 | -0.14 | 0.02 |
| **Peaches** | 0.06 | 0.07 | 0.02 | 0.04 | 0.01 | 0.08 | **0.44** | **0.56** |
| **Peanut butter** | 0.13 | 0.27 | 0.19 | 0.20 | -0.01 | -0.02 | -0.01 | 0.04 |
| **Pears** | -0.03 | -0.11 | 0.05 | 0.18 | 0.07 | 0.03 | **0.36** | 0.29 |
| **Peas** | 0.22 | 0.22 | -0.08 | -0.03 | **0.55** | **0.53** | -0.16 | -0.06 |
| **Pineapple** | 0.15 | 0.16 | -0.04 | 0.07 | 0.02 | 0.08 | **0.43** | **0.38** |
| **Pizza** | **0.38** | **0.44** | 0.16 | 0.09 | 0.08 | 0.03 | -0.12 | 0.01 |
| **Polyunsaturated margarine** | 0.06 | 0.08 | 0.08 | -0.01 | -0.08 | -0.04 | -0.05 | -0.09 |
| **Pork** | **0.43** | **0.34** | -0.03 | -0.18 | -0.02 | 0.14 | 0.15 | 0.30 |
| **Porridge** | -0.02 | 0.09 | 0.20 | 0.13 | 0.11 | 0.13 | 0.02 | 0.17 |
| **Potatoes** | 0.30 | 0.32 | -0.05 | -0.07 | **0.40** | **0.47** | -0.20 | -0.08 |
| **Pumpkin** | 0.18 | 0.14 | 0.05 | 0.10 | **0.55** | **0.48** | -0.04 | -0.04 |
| **Reduced fat milk** | 0.04 | -0.06 | -0.03 | 0.03 | -0.17 | -0.17 | -0.05 | -0.02 |
| **Rice** | 0.16 | 0.28 | **0.38** | **0.31** | 0.04 | -0.02 | 0.04 | 0.06 |
| **Ricotta or cottage cheese** | 0.05 | 0.00 | 0.21 | 0.19 | -0.07 | -0.05 | 0.10 | 0.06 |
| **Rye bread** | -0.10 | -0.05 | 0.08 | 0.15 | 0.07 | -0.15 | 0.16 | 0.01 |
| **Salami** | **0.41** | **0.32** | -0.02 | -0.10 | 0.08 | -0.03 | 0.01 | 0.14 |
| **Sausages** | **0.49** | **0.53** | 0.04 | -0.13 | 0.11 | 0.17 | -0.02 | -0.02 |
| **Skim milk** | -0.05 | -0.06 | 0.02 | 0.07 | -0.03 | -0.09 | 0.23 | 0.10 |
| **Soft cheese** | 0.13 | 0.10 | 0.17 | 0.12 | -0.06 | -0.06 | 0.03 | -0.08 |
| **Soya milk** | -0.18 | -0.09 | 0.28 | 0.20 | 0.15 | -0.01 | 0.04 | 0.07 |
| **Spinach** | 0.06 | 0.05 | **0.35** | **0.41** | **0.41** | **0.28** | 0.18 | 0.23 |
| **Spirits** | 0.14 | 0.15 | -0.01 | 0.00 | 0.05 | 0.03 | -0.04 | -0.09 |
| **Strawberries** | 0.08 | 0.15 | 0.04 | 0.06 | 0.07 | -0.07 | **0.40** | **0.40** |
| **Sugar** | 0.19 | 0.08 | -0.18 | -0.18 | 0.08 | 0.26 | -0.16 | -0.01 |
| **Sweet biscuits** | **0.32** | **0.34** | 0.07 | 0.09 | -0.05 | 0.05 | 0.06 | -0.03 |
| **Tinned fish** | 0.16 | 0.17 | 0.21 | 0.19 | 0.09 | -0.09 | 0.25 | 0.20 |
| **Tinned fruit** | 0.09 | 0.19 | 0.06 | 0.03 | 0.04 | 0.15 | 0.13 | 0.26 |
| **Tofu** | -0.20 | -0.11 | **0.44** | **0.37** | 0.08 | 0.01 | 0.06 | 0.01 |
| **Tomatoes** | -0.09 | -0.03 | **0.37** | **0.27** | -0.05 | -0.04 | -0.04 | 0.16 |
| **Tomato sauce** | 0.24 | 0.31 | 0.25 | 0.09 | 0.16 | 0.11 | -0.17 | 0.01 |
| **Veal** | 0.19 | 0.21 | -0.02 | -0.09 | 0.08 | -0.02 | 0.09 | 0.18 |
| **Vegemite** | 0.29 | 0.36 | 0.13 | 0.03 | 0.03 | 0.11 | 0.03 | 0.03 |
| **Weet-Bix** | 0.04 | 0.11 | 0.02 | -0.01 | 0.12 | 0.19 | 0.03 | 0.01 |
| **White bread** | 0.26 | 0.08 | -0.23 | -0.31 | 0.05 | 0.12 | -0.15 | -0.14 |
| **Wholemeal bread** | -0.09 | 0.03 | 0.10 | 0.14 | 0.01 | 0.02 | 0.05 | -0.03 |
| **Yoghurt** | 0.05 | 0.01 | 0.17 | 0.28 | -0.10 | -0.06 | **0.35** | **0.25** |
| **Zucchini** | -0.04 | 0.10 | **0.50** | **0.44** | **0.39** | **0.36** | 0.07 | 0.02 |
| **Red wine** | 0.01 | 0.04 | 0.29 | 0.28 | -0.18 | -0.22 | -0.01 | -0.08 |
| **White wine** | 0.07 | 0.07 | 0.17 | 0.10 | -0.14 | -0.22 | 0.06 | -0.02 |
| **Light beer** | 0.04 | 0.02 | 0.01 | -0.01 | -0.08 | -0.07 | -0.04 | -0.04 |
| **Heavy beer** | 0.11 | 0.08 | 0.26 | 0.19 | -0.08 | -0.07 | -0.03 | -0.13 |

^a^ Values are correlation coefficients between each food item and the dietary pattern derived from factor analysis. Factor loadings above 0.30 are shown in bold. ^b^ Other beans: chickpeas, lentils, etc.

**Supplementary Table 5.** Change of weight and energy intake from preconception to during pregnancy.

| Variables | Time points  (n= 621) | Mean (SD) | Spearman’s correlation coefficient (r) |
| --- | --- | --- | --- |
| Weight (kg) | Before pregnancy  During pregnancy  Mean difference | 65.79 (13.32)  68.48 (14.07)  2.69 (7.04) | 0.87 |
|  | *p-value ^b^* | < 0.0001 | < 0.0001 |
| Total energy intake (KJ/day) | Before pregnancy  During pregnancy  Mean difference | 6487.64 (2259.48)  7135.74 (2160.63)  648.09 (2270.39) | 0.46 |
|  | *p-value ^b^* | < 0.0001 | < 0.0001 |

^a^ Values are mean (SD) or correlation coefficients (r), ^b^ *p-values* from the paired t-test, Spearman’s correlation.
